# DNA modifications of Durham Collection phages and promiscuity of GmrSD-family Type IV restriction enzyme BrxU

**DOI:** 10.1128/aem.00810-26

**Published:** 2026-06-12

**Authors:** Jennifer J. Readshaw, Abigail Kelly, Yan-Jiun Lee, Giuseppina Mariano, Liam P. Shaw, Peter R. Weigele, Tim R. Blower

**Affiliations:** 1 Department of Biosciences, Durham University 3057, Durham, United Kingdom; 2 New England Biolabs 1696 , Ipswich, Massachusetts, USA; 3 School of Infection and Immunity, University of Glasgow 3526, Glasgow, United Kingdom; 4 Department of Biology, University of Oxford 6396, Oxford, United Kingdom; University of Nebraska-Lincoln, Lincoln, Nebraska, USA

**Keywords:** bacteriophage, DNA modification, Durham, restriction enzyme, GmrSD, phage defense

## Abstract

Bacteriophages (phages), viral predators of bacteria, are an attractive way to combat the rise of antimicrobial resistance. By infecting and killing bacteria, phages generate selection pressure for the evolution of defense systems. Successfully applying phages in the clinic will, in part, depend on understanding and predicting how bacterial defense systems determine the outcomes of a phage infection. Here, we present morphological, genomic, phylogenetic, and modification-based characterization of 12 new bacteriophage species targeting *Escherichia coli*, isolated from water sources in Durham, UK, during undergraduate practical classes. These phages, added to our growing “Durham Collection,” were all determined to be sensitive to the GmrSD-family Type IV restriction enzyme, BrxU. As such, these phages have modified genomic DNAs. HPLC and MS analysis of the genomic DNAs identified a range of modifications present in the *Tequatrovirus*, *Krischvirus*, and *Mosigvirus* phages, the latter of which contained 5-arabinosyl-2′-deoxycytidine (5-ara-dC) and disaccharide arabinobiose (5-ara-ara-dC) moieties. Curiously, *Krischvirus* phages were shown to have modification pathways distinct from those of *Tequatrovirus* phages. Finally, testing the modified genomic DNAs in *in vitro* cleavage assays with BrxU demonstrated cleavage of all modifications tested. This further extends the broad substrate specificity previously identified for BrxU. Collectively, these data provide a larger standardized Durham Collection to be used for better prediction of phage-host interactions and infection outcomes.

**IMPORTANCE:**

Widespread antibiotic use has led to rising rates of antibiotic resistance. It is estimated that deaths from antibiotic-resistant bacterial infections will outpace deaths from cancer by 2050. Alternate methods of treatment are required. Bacteriophages (phages), are viruses that specifically target bacteria and are predominantly harmless to humans. There is increased interest in using phage therapy in clinics to treat infections. Studying interactions between bacteria and phages is necessary so that we can understand and better predict the outcomes of phage therapy. This will increase the chances of clinical success. Our presented work provides detailed characterization of a set of phages isolated from the environment that infect *Escherichia coli*, a common pathogen and model experimental system. Standardized collections of phages are time-consuming to generate and the results from our ongoing characterization of the Durham Collection presented here represent a community resource for the ease of comparison between these and other phages strains, as well as across different experimental systems.

## INTRODUCTION

In recent years, there has been renewed interest in the study of interactions between bacteria and their viral parasites, bacteriophages, due to the potential applications in biotechnology and in the clinic. Many of our revolutionary biotechnological tools are derived from cellular defenses evolved as a result of selection pressures from phage predation, such as restriction-modification systems (1), abortive infection systems (2–4), and CRISPR-*cas* (5). There has since been a rapid expansion in the number of new bacterial phage defense systems discovered owing to the increased availability and application of sequencing technologies, high-throughput screening, and comparative genomics. These newly discovered systems can often be diverse (6–12). Many defense systems have protein domains that show evidence of sequence and structural conservation (13) but might be divergent in function. Defense systems appear to cluster into genetic islands (14) and likely form multilayered defense networks (15). There is evidence of coordinated regulation of defenses, as demonstrated by the widespread BrxR WYL-domain transcriptional repressors (16–18) and RptR repressor families (19). Bacteria arguably possess an immune system that is both analogous and indeed, in some cases, homologous to mammalian immune systems or components (20).

Phages are being studied for applications in the clinic, using “phage therapy” as an alternate means to treat antibiotic-resistant bacterial infections (21, 22). Successful treatment will rely upon selecting the correct combination of phages with sufficient host range and ability to evade bacterial phage defenses. Machine learning has been used to predict the outcomes of phage-host interactions based on genomics (23). Coupling genomic and phenotypic data will further enhance our predictive capabilities and will be dependent on the use of well-characterized phage collections. The ongoing and very productive SEA-PHAGES program catalogs mycobacterial phages (24) and has already provided phages that were successfully deployed in the clinic (25). Recently, the BASEL collection, another excellent resource for coliphage research (26), was also updated with additional phage families for greater taxonomic coverage (27). We recently established the Durham Collection, composed of environmental coliphages isolated by undergraduates as part of their laboratory curriculum (28). Those initial phages establishing this collection were characterized at the genomic, phylogenetic, and morphological levels and include an entirely new genus of phage (28).

The first batch of the Durham Collection was also used to study Bacteriophage Exclusion (BREX) phage defense systems (9, 14, 29–32) and to correlate BREX recognition sequences in phage genomes with the veracity of defense (28). We previously demonstrated that BREX and a GmrSD homolog, BrxU, encoded within a defense island on a multidrug-resistant plasmid of *Escherichia fergusonii*, provide complementary phage defense (14). BREX acts on non-modified DNA (14). In contrast, BrxU is a Type IV restriction enzyme that has broad substrate recognition, cleaving DNA containing a range of modifications at the C5 position of cytosine (14, 33). As part of this earlier study, we isolated a set of phages that were sensitive to BrxU and not BREX, implying these phages have modified genomes (14). For example, the T-even phages T2, T4, and T6 have the modified nucleoside 5-hydroxymethyl-2′-deoxycytidine (5-hmdC) and its glycosylated derivatives in their DNA (34). The range of known modified (35) and hypermodified (36) bases is rapidly increasing, with modifications identified on nucleotides dG, dA, dC, and dT (37, 38), and some phages can fully replace dT with dU (39). Although DNA modifications can protect injected phage DNA from being targeted by some defense systems, including restriction enzymes (40), BREX (41), and CRISPR-*cas* (42), they can be targeted by Type IV (modification-dependent) restriction enzymes (43).

Here, we present the characterization of 12 phages newly added to the Durham Collection. All phages have modified bases in their genomic DNA and have been fully characterized by genome sequence, domain-level annotation, virion morphology, and nucleoside composition. They include *Tequatrovirus* phages, a *Mosigvirus*, and a *Krischvirus*. Identification of the DNA modifications separated the phages into four groups, including *Mosigvirus* NP that features the recently characterized monosaccharide 5-arabinosyl-2ʹ-deoxycytidine (5-ara-dC) and disaccharide “arabinobiose” (5-ara-ara-dC) modifications (44, 45). Testing *in vitro* cleavage activity of BrxU on DNA substrates containing these modifications demonstrated that BrxU has even broader substrate specificity than previously identified (14, 46). Future use of the Durham Collection will allow similar detailed examination of phage-host interactions and aid efforts to successfully use phage therapy in clinics.

## RESULTS

### Characterization of Durham Collection coliphages restricted by BrxU *in vivo*

We previously identified a series of coliphages that were susceptible to BrxU (14), a homolog of the GmrSD Type IV (modification-dependent) restriction enzymes (47). The phages were isolated from water sampled around Durham City, UK, as part of an undergraduate practical course in 2016 and 2017, as described previously (28). Based upon plaque assays, BrxU provided protection against 13 phages from our growing Durham Collection (14, 28). As BrxU cleaves DNA containing modified cytosines (14), we hypothesized that the 13 phages all had genome modifications. We aimed to characterize these phages and determine the DNA modifications present, as this information will enrich the utility of the Durham Collection and enable us to learn more about BrxU substrate preferences.

Following next-generation sequencing of the 13 genomes, there was one phage, TB36, that proved identical to TB37 and so was excluded from further characterization. Phage metadata are summarized in Table 1, with additional details provided in Table S1. We used CheckV v1.0.1 (48) to assess the quality of the phage genomes using the full pipeline (end_to_end). All 12 phage genomes showed no contamination, had high completeness scores (range: 95.5%–100%, *n* =4 with 100% completeness; see Table S2), and were graded as high quality per the Minimum Information about an Uncultivated Virus Genome standard (49). The internal CheckV quality of the genomes was at least “high quality” (*n* = 10), with some assessed as “complete” (*n* = 2, BGP and Solly). These 12 genome sequences were annotated and submitted to GenBank. While each phage was given its own unique scientific name on submission to the European Nucleotide Archive (ENA), we use the abbreviated names throughout this article for ease of reference (Table 1 and Table S1).

**TABLE 1:** Summary information for 12 Durham Collection phages with modified DNA

Phage	AKA	Genome size (bp)	ORFs (no.)	tRNAs (no.)	GC (%)	Taxonomic group	GenBank accession no.	Closest relative	Coverage (%)	Identity (%)	GenBank accession no.
*Escherichia* phage vB_Eco_BGP	BGP	168,661	288	10	35	*Tequatrovirus*	OZ035849.1	*Yersinia* phage phiD1	91	97.20	NC_027353.1
*Escherichia* phage vB_Eco_CP	CP	166,864	277	11	35	*Tequatrovirus*	OZ034683.1	*Escherichia* phage UTI-E4	94	96.47	OL870317.1
*Escherichia* phage vB_Eco_EH2	EH2	160,527	273	11	35	*Tequatrovirus*	OZ034684.1	*Escherichia* phage Teqhal	97	98.63	MN895435.1
*Escherichia* phage vB_Eco_EL	EL	167,504	286	10	35	*Tequatrovirus*	OZ034682.1	*Escherichia* phage JK1	95	97.65	MZ436830.1
*Escherichia* phage vB_Eco_Geo	Geo	167,505	292	10	35	*Tequatrovirus*	OZ034685.1	*Escherichia* phage vB_EcoM_Lutter	94	97.52	NC_054924.1
*Escherichia* phage vB_Eco_NicPhage	NP	170,265	285	2	38	*Mosigvirus*	OY726582.1	*Escherichia* phage AlbertHofmann strain Bas47	93	96.59	MZ501047.1
*Escherichia* phage vB_Eco_NR1	NR1	167,153	260	10	35	*Tequatrovirus*	LR990704.1	*Shigella* phage CM8	92	96.83	NC_054939.1
*Escherichia* phage vB_Eco_QOTSP	QOTSP	168,413	287	10	35	*Tequatrovirus*	OY720014.1	*Escherichia* phage Teqskov	93	97.30	NC_054934.1
*Escherichia* phage vB_Eco_SAP	SAP	165,332	290	0	41	*Krischvirus*	OZ034687.1	*Escherichia* phage vB_Eco_TB34	100	99.96	OX001802.1
*Escherichia* phage vB_Eco_Solly	Solly	166,664	283	11	37	*Tequatrovirus*	OY720021.1	*Escherichia* phage Teqsoen	97	96.96	MN895436.1
*Escherichia* phage vB_Eco_Some	Some	167,255	283	11	35	*Tequatrovirus*	OZ034686.1	*Escherichia* phage Teqsoen	96	97.50	MN895436.1
*Escherichia* phage vB_Eco_TB37	TB37	166,495	222	11	36	*Tequatrovirus*	OZ035850.1	*Escherichia* phage Teqdroes	96	98.26	MN895438.1

**Fig 1:**
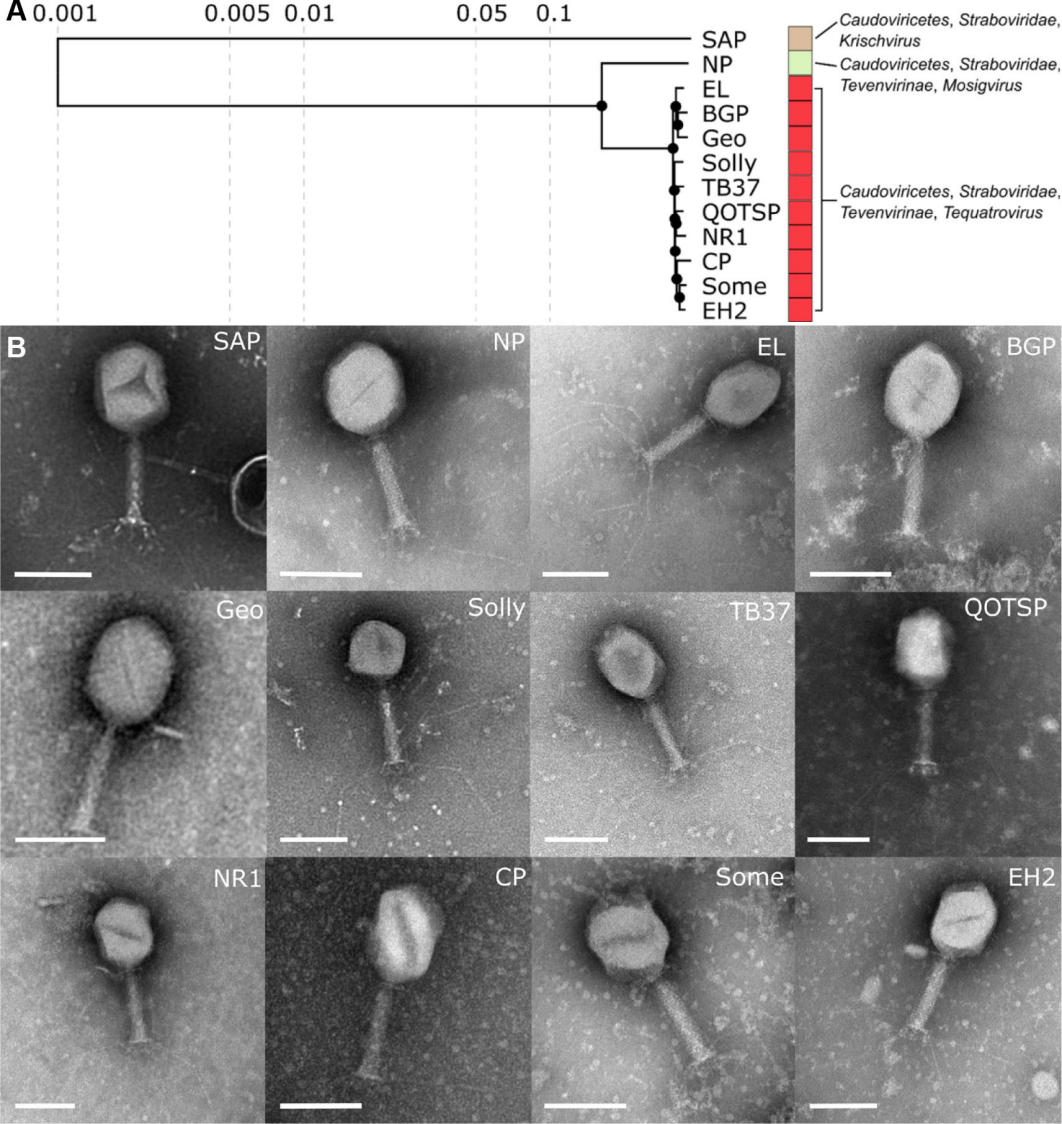
Durham Collection phages with DNA modifications represent three genera. (**A**) Phylogenetic analysis shows relatedness between phages; the scale represents the genomic distance score (SG) calculated by tBLASTx. (**B**) Electron micrographs of target phages. Scale bars represent 100 nm.

The genomes range in size from 160,527 to 170,265 bp. The obtained genomes were used for comparison against known phages in the database and to examine relatedness within the collection through the generation of a phylogenetic tree (Fig. 1A). This subset of the Durham Collection includes phages that are all part of class *Caudoviricetes* and family *Straboviridae*. Phage SAP is a representative of the genus *Krischvirus*, and phage NP is part of the subfamily *Tevenvirinae* and the genus *Mosigvirus* (Fig. 1A). The 10 remaining phages are all more closely related, being part of the subfamily *Tevenvirinae* and the genus *Tequatrovirus*, which also includes T2, T4, and T6 (Fig. 1A). Taxonomies were determined as per the current ICTV taxonomy release (https://ictv.global/taxonomy). Within the 10 *Tequatrovirus* phages, BGP and Geo are closely related (95% coverage, 98.67% shared sequence identity) and are then also closely related to EL (95% coverage, 98.42% shared sequence identity between BGP and EL; 95% coverage, 98.87% shared sequence identity between Geo and EL). Phages Some and EH2 are closely related, sharing 97.85% sequence identity (94% coverage). Comparing this second batch of genomes with our first batch of Durham Collection phages (28), we noted that phages SAP (from this batch of Durham phages) and TB34 (from our previous batch of Durham phages) share 99.96% sequence identity (100% coverage). Notably, in previous phage plaquing tests, SAP was susceptible to BrxU, but TB34 was not (14).

Transmission electron microscopy was then performed to visualize phage virions (Fig. 1B). All 12 phages were imaged with full capsids, and we observed clear rigid tails and baseplates, indicating a myoviral morphology (Fig. 1B). Every phage had a prolate head, with the exception of Solly, which had a more isometric head morphology. In some instances, it was possible to see details of tail fibers, especially in the cases of phages EL, Solly, and TB37, though some evidence of tail fibers was visible for all samples (Fig. 1B). Overall, the phages were similar in their morphologies. Collectively, these data show genomic and morphological characterization of this second cohort of coliphages from the Durham Collection.

### Diversity of the Durham Collection

**Fig 2:**
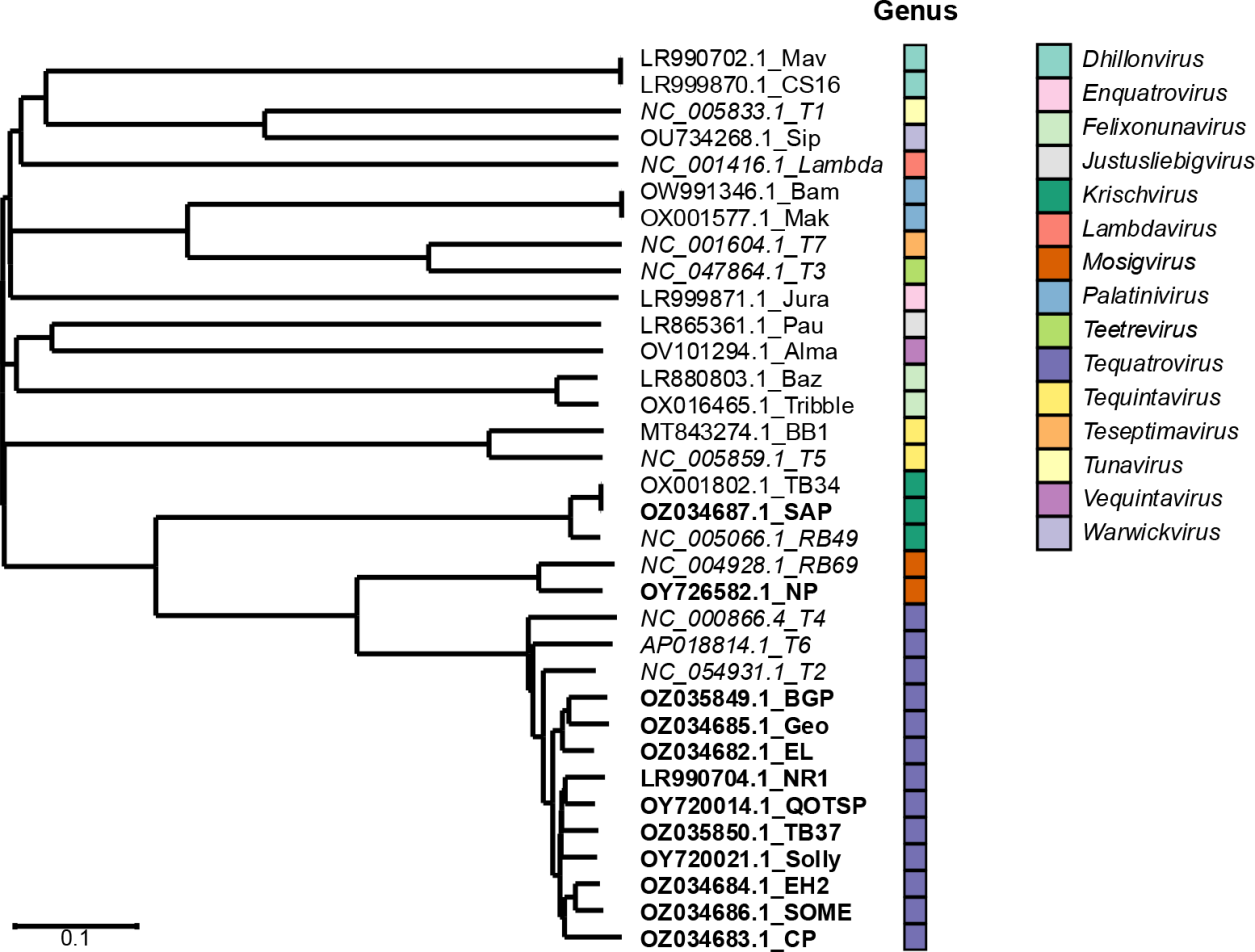
Comparative taxonomy of all 24 Durham Collection phages. The new additions with modified genomes are shown in bold. Previously published Durham Collection phages are shown in regular text. For context, classic *Caudoviricetes* phages T1–T7, Lambda, RB49, and RB69 are also shown in italics. All are provided with accession numbers. The tree scale reported refers to the branch length metadata of the tree. Colored boxes indicate the taxonomy of each branch at the genus level.

Following the addition of the 12 additional phages (Fig. 1), we aimed to examine taxonomy across the Durham Collection. The resulting phylogenetic tree includes the full 24 phages of the Durham Collection alongside common coliphages T1–T7, Lambda, RB49, and RB69 to provide ease of context (Fig. 2). This demonstrates the taxonomic diversity across the Durham Collection.

Next, we wanted to more closely examine the taxonomic distribution of the 12 modified phages. To define the phylogenetic relationship between the modified Durham phages and the broader NCBI database, we first used mash dist (mash v2.3) to compare the modified Durham phages against the complete NCBI RefSeq genomes (using the sketch database, https://gembox.cbcb.umd.edu/mash/refseq.genomes.k21s1000.msh, on 6 August 2023) (50). The resulting top hits for all 12 Durham phages were part of the *Straboviridae* family (Table S3). To contextualize these Durham phages relative to other *Straboviridae* phages, we used VIPtree (51) to build phylogenies based on proteomes. First, we employed fastANI v1.33 and ANIcluster (see Materials and Methods) to eliminate redundancy and reduce our NCBI data set to ≤200 genomes for submission on VIPtree (51). We then retrieved the taxonomic information of the obtained genomes using the Entrez Suite v16.2 (Table S4). The resulting VIPtree phylogenetic tree for this curated set of *Straboviridae* genomes demonstrates that the majority, 10 of 12 Durham coliphages in this study, cluster with classic coliphages T2, T4, and T6 within *Tequatrovirus* (Fig. S1). NP, as part of *Mosigvirus*, is related to phage RB69, which was characterized as having an arabinose-derived DNA modification, tentatively reported as arabinosylated 5-hmdC (52) (Fig. S1). SAP (from this batch of Durham phages) and TB34 (from our previous batch of Durham phages) also clustered together within the genus *Krischvirus*, which includes pseudo-T-even phages such as RB49 (53).

### Durham Collection phages contain diverse cytosine modifications

**Fig 3:**
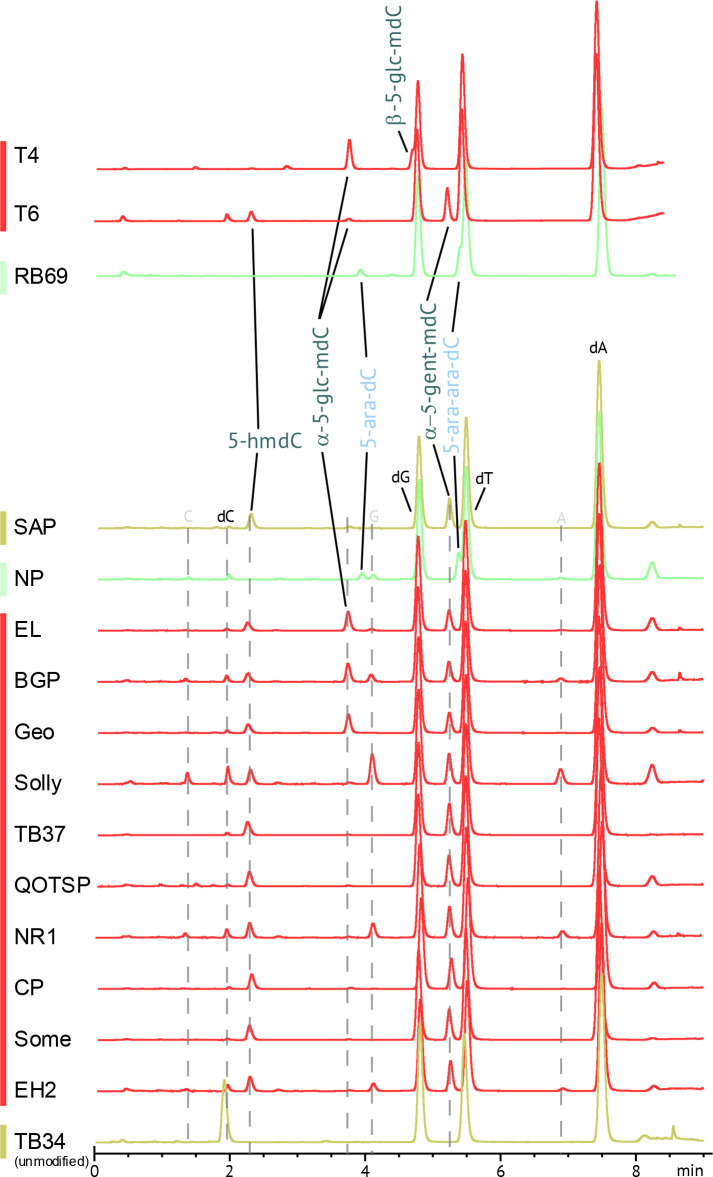
Modified Durham collection phages have diverse cytosine modifications. Analysis of phage genomic DNA by digestion and HPLC/MS identified modifications. Modifications: 5-hydroxymethyl-2′-deoxycytidine (5-hmdC), α−5-glucosylmethyl-2′-deoxycytidine (α−5-glc-mdC), α−5-gentiobiosylmethyl-2′-deoxycytidine (α−5-gent-mdC), 5-arabinosyl-2′-deoxycytidine (5-ara-dC), and disaccharide arabinobiose (5-ara-ara-dC).

We then determined the identity of the DNA modifications present in each of the 12 phage genomes through a process of enzymatic hydrolysis, HPLC separation, and mass spectrometry (MS) (54). As TB34 is related to SAP but shows no BrxU susceptibility, we also examined the DNA of TB34, with the hypothesis that it would be unmodified. Phages T4, T6, and RB69 were also analyzed in order to provide molecular standards for comparison. Extracted phage genomic DNAs were first enzymatically hydrolyzed to free nucleosides, and the resulting mixtures were then separated by HPLC with masses from each nucleoside determined using MS as described. The chromatograms for each phage sample yielded peaks corresponding to a diverse set of non-canonical nucleosides across the collection (Fig. 3). As hypothesized, TB34 did not have detectable modified DNA (Fig. 3). All 12 of our BrxU-susceptible phages did have modifications, and these were all cytosine derivatives. We detected 5-hydroxymethyl-2′-deoxycytidine (5-hmdC) and α−5-glucosylmethyl-2ʹ-deoxycytidine, where the glucose is attached to the C5 methyl via an α-linkage (α-5-glc-mdC), but not the β-glucosylated form (Fig. 3). Linkage was confirmed by comparison against T4, which contains both linkages (Fig. S2). These known modifications of T4-like phages were consistent with our phylogenetic clustering (Fig. 2 and Fig. S1). We also detected gentiobiose, a disaccharide composed of two glucose units coupled by a β(1- > 6) linkage, attached to a cytosine C5 methyl via an α-linkage. This cytosine derivative α−5-gentiobiosylmethyl-2ʹ-deoxycytidine (α−5-gent-mdC), is absent from T4 but found replacing about 5% of T2 cytidines and 72% of T6 cytidines (55), again matching our phylogenetic clustering. Phage NP contained an arabinose modification of cytosine (Fig. 3). Whereas related phage RB69 was erroneously identified as having a methyl group within a larger modification arabinosyl-5-hydroxymethyl-2ʹ-deoxycytidine (44, 45, 52, 56), phage NP is unmethylated, with the arabinose sugar linked directly to the C5 of the cytosine nucleobase, forming 5-ara-dC (Fig. 3). Furthermore, NP also had 5-ara-ara-dC modifications (Fig. 3). Finally, we also note that ribonucleotides were observed in some samples. Though we cannot rule out ribonucleotides from being incorporated in the viral DNA, as ribonucleotides frequently co-purify with phage DNA we consider them more likely to be artifacts of purification.

**Fig 4:**
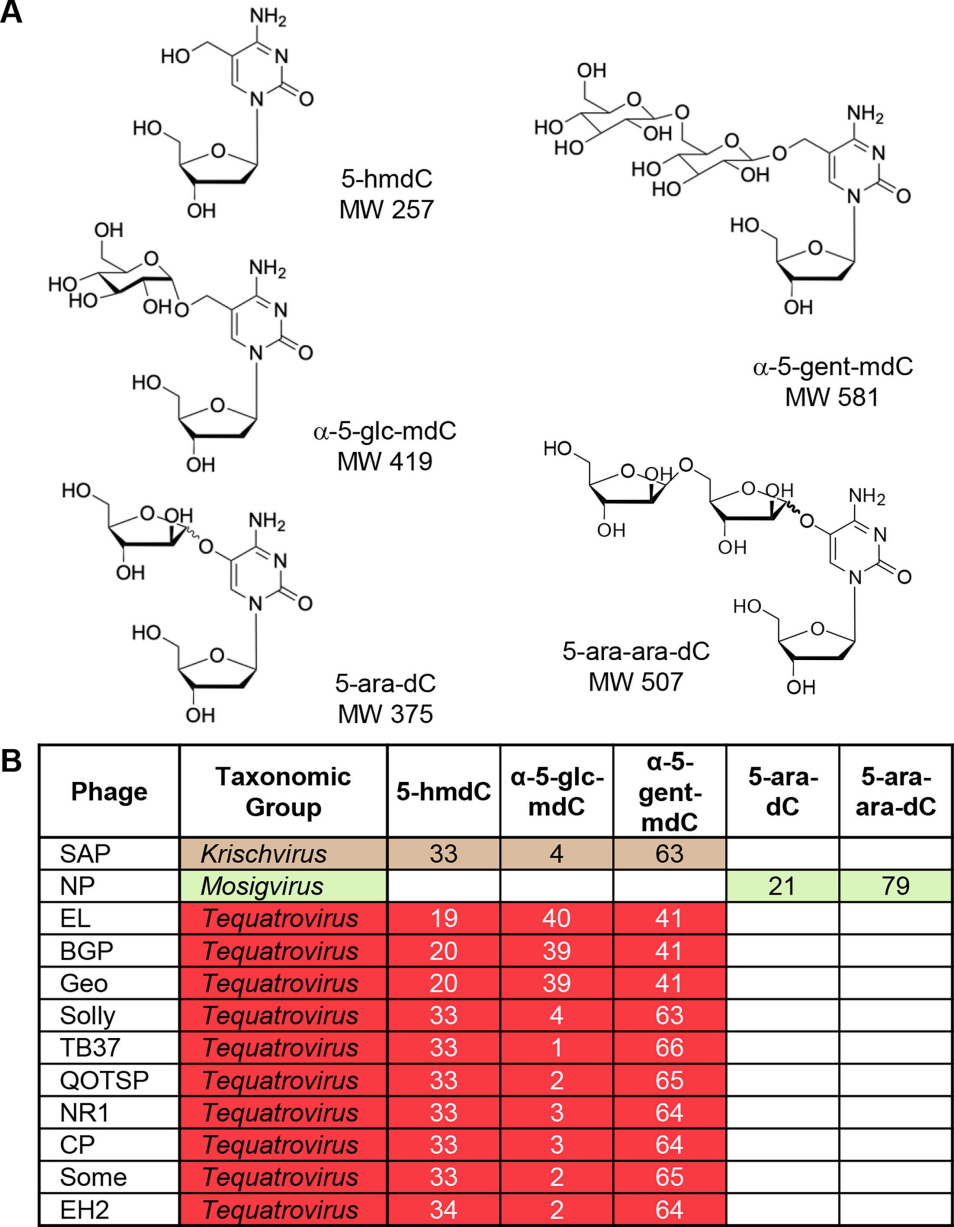
Clustering of Durham Collection phages by DNA modification. (**A**) Structures of observed modified nucleosides. (**B**) Modified Durham phages cluster into four groups based on phylogeny and modification: (i) SAP; (ii) NP; (iii) EL, BGP, and Geo; and (iv) Solly, TB37, QOTSP, NR1, CP, Some, and EH2. Modifications: 5-hydroxymethyl-2′-deoxycytidine (5-hmdC), α−5-glucosylmethyl-2′-deoxycytidine (α−5-glc-mdC), α−5-gentiobiosylmethyl-2′-deoxycytidine (α−5-gent-mdC), 5-arabinosyl-2′-deoxycytidine (5-ara-dC), and disaccharide arabinobiose (5-ara-ara-dC).

The phages could then be clustered according to DNA modifications (Fig. 4A), which loosely followed patterns in the phylogenetic tree (Fig. 1A and 4B). Of the phages belonging to the genus *Tequatrovirus*, EL, BGP, and Geo genomic DNA contained three modifications—5-hmdC, α−5-glc-mdC, and α−5-gent-mdC—in an approximate 1:2:2 ratio, respectively (Fig. 4B). Though from the same *Tequatrovirus* genus, genomic DNA from Solly, TB37, QOTSP, NR1, CP, Some, and EH2 contained ~one-third 5-hmdC, ~two-thirds α-5-gent-mdC, and a low background level of α-5-glc-mdC (Fig. 4B). SAP could also be grouped together with these seven phages by modification, though SAP is phylogenetically distinct (Fig. 4B and Fig. 1A). NP was the single phage for which the genomic DNA contained mono- and di-arabinosylated cytidines, present in a 1:4 ratio (Fig. 4B).

**Fig 5:**
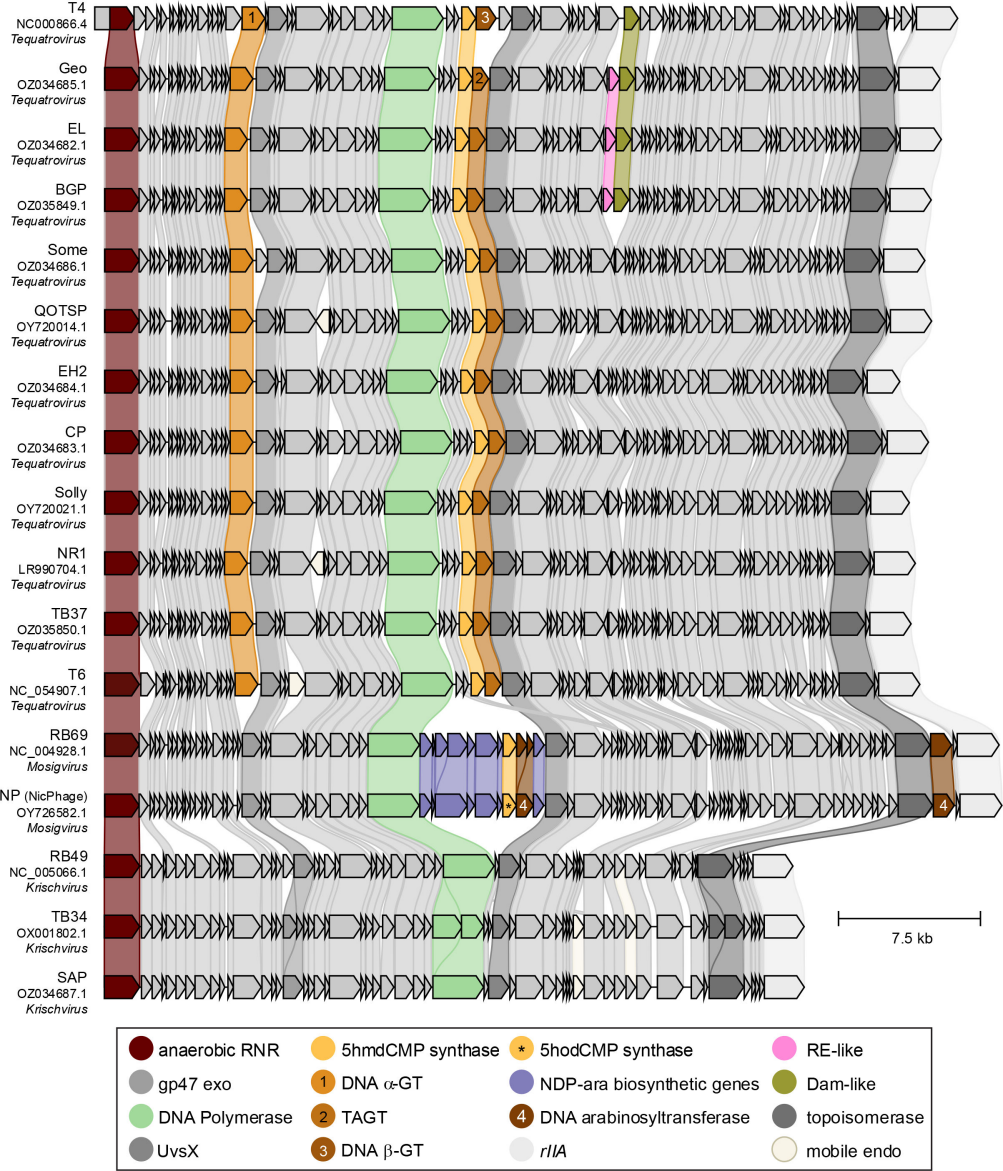
Genomic comparisons of the DNA modification gene neighborhoods. Subgenomic regions encompassing DNA modification pathway genes and spanning the anaerobic ribonucleotide reductase gene (nrdD) to the *rIIA* gene from 12 of the Durham Collection phages targeted by BrxU, TB34, and four representative *Straboviridae* (T4, T6, RB49, and RB69) were aligned using Clinker. Genes encoding functions for DNA modification, together with landmark genes, are colored and indicated in the key below the alignments. Gene boxes are drawn to scale. GT, glycosyltransferase; TAGT, Tet-associated glycosyltransferase.

Examining the genomes of representatives from each modification grouping, we can postulate the genes required to make the observed modifications (Fig. 5). Selecting a region of each genome bordered by anaerobic ribonucleotide reductase and *rIIA*, then performing an alignment using Clinker (57), helped to identify genetic synteny between the modification-producing genes of each of the four clusters (Fig. 4 and 5). The Durham *Tequatrovirus* phages are highly syntenic with T6 and T4. Like T6, it encodes a DNA α-glucosyltransferase and a “Tet-associated glycosyltransferase” (TAGT, Pfam: PF2069) likely responsible for synthesis of di-glucosylated cytosines by addition of a second glucose to α-5-glc-mdC (Fig. 5). They do not, however, encode DNA β-glucosyltransferase (DNA β-GT), which is found in T4 at the position syntenic with TAGT (Fig. 5). T4 DNA therefore exclusively contains mono-glucosylated bases comprising a mixture of α- and β-linked stereoisomers (Fig. S2). The absence of genes encoding DNA β-GT in the Durham phages characterized here could explain why β-5-glc-mdC was not detected among their DNAs (Fig. 3 and Fig. S2). Geo, EL, and BGP differ from the other Durham *Tequatrovirus* phages in the examined region through the presence of a Dam-like gene shared with T4 and a restriction enzyme-like gene exclusive to only Geo, EL, and BGP (Fig. 5).

Like the *Mosigvirus* RB69, phage NP DNA contains a mixture of mono- and di-arabinosylated 2′-deoxycytidines (Fig. 5). Whereas *Tequatrovirus* phages glucosylate 5-hydroxymethyl-2′-deoxycytidine, the modified cytidines of *Mosigvirus* are derived by the arabinosylation of 5-hydroxy-2′-deoxycytidine, such that the sugar moiety is connected directly to the pyrimidine ring via an ether linkage at C5. The precursor, 5-hydroxy-2′-deoxycytidine, is synthesized at the mononucleotide stage by a 5-hydroxy-2′-deoxycytidine monophosphate synthase (Fig. 5; 5-hodCMP synthase, indicated by “*”), an enzyme distinct in its activity but homologous to 5-hydroxymethyl-2′-deoxycytidine synthase. Both enzymes are members of the thymidylate synthase superfamily. *Mosigvirus* encodes two putative DNA arabinosyltransferases hypothesized to sequentially install arabinosyl moieties. The first is located within a cluster of putative sugar-modifying genes (Fig. 5, indicated in light purple), including a predicted isomerase, epimerase, sugar kinase, and NTP-transferase that might produce the arabinose precursor for transfer; the other is located between *rIIA* and a topoisomerase gene (Fig. 5).

Phages TB34 and SAP belong to the genus *Krischvirus* and reveal a curious and as yet unexplained phenomenon. Despite being greater than 99% identical to each other at the nucleotide sequence level, TB34 genomic DNA contains canonical cytosines (Fig. 3), whereas SAP contains a full complement of cytosine modifications (Fig. 3). As such, TB34 is immune to BrxU cleavage, but SAP is cleaved (14). The genetic and biochemical mechanisms whereby SAP synthesizes glucosylated methyl-2′-deoxycytidines are unknown, as genomes of *Krischvirus* lack homologs of 5hmCMP and 5hodCMP synthases. Additionally, genes encoding DNA glucosyltransferases have not been detected. These differences with other members of the *Straboviridae* are reflected at the neighborhood level; the genes for 5-hmdC synthesis and DNA glycosylation are absent relative to the syntenic locations in *Tequatrovirus* and *Mosigvirus*. As SAP and TB34 are closely related (99.96% sequence identity with 100% coverage), we performed a SNIPPY (https://github.com/tseemann/snippy) analysis to map SNPs and attempted to identify candidate genes responsible for the genome modifications in SAP (Fig. S3). Unfortunately, this analysis failed to indicate any obvious leads. Determining the modification pathway for *Krischvirus* will require a separate study.

### BrxU cleaves DNA with a broad range of cytosine modifications

**Fig 6:**
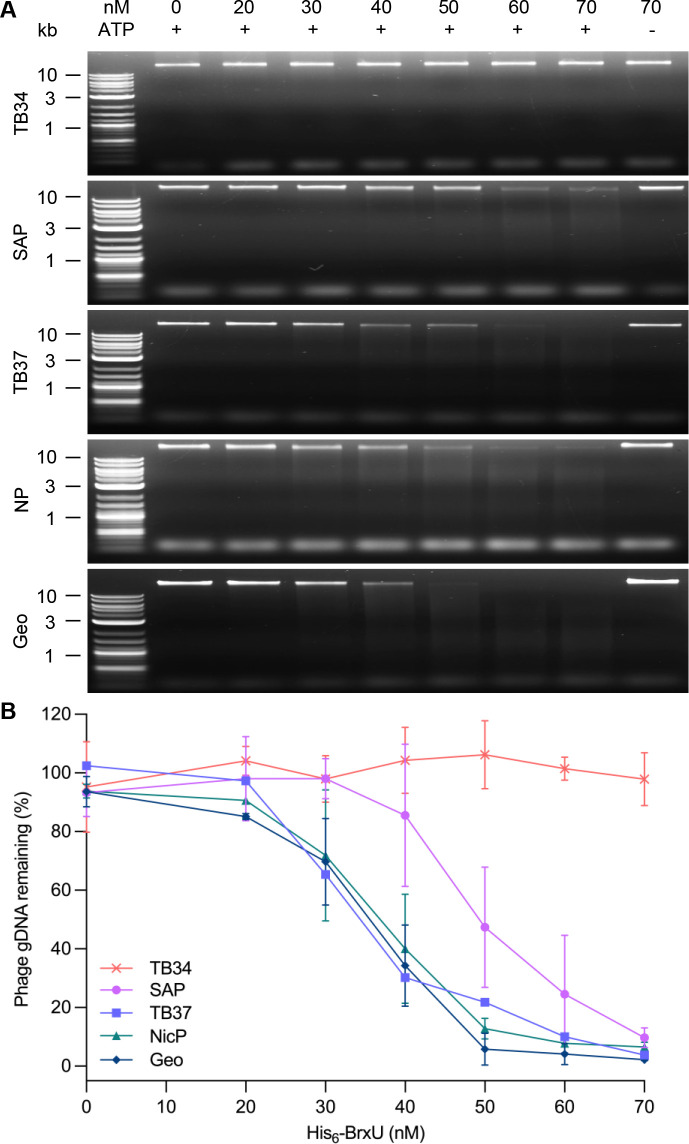
Digestion of Durham Collection phage genomes by His_6_-BrxU. (**A**) Purified TB34, SAP, TB37, NP, and Geo gDNA at 300 ng were incubated with His_6_-BrxU at 0–70 nM in the presence (+) or absence (−) of ATP for 10 min and resolved on 0.8% agarose gels. Reactions were performed in triplicate, and representative gels are shown. (**B**) The average percentage of phage gDNA remaining in reactions with ATP compared to reactions without ATP, calculated from triplicate gels. Error bars represent standard deviations.

Having determined the modifications on the phage genomic DNAs, we used the varied DNA as substrates for *in vitro* cleavage by BrxU to investigate substrate preferences. His_6_-BrxU was expressed and purified, then titrated and incubated with phage genomic DNAs in the presence or absence of ATP. Resulting products were analyzed by agarose gel electrophoresis and quantified (Fig. 6A and B). TB34 was used as a non-modified DNA control and was not digested (Fig. 6A and B). His_6_-BrxU had the least impact on genomic DNA from SAP, while genomic DNAs from TB37, NP, and Geo were preferred and cut similarly (Fig. 6A and B). In the absence of ATP, no digestion was observed (Fig. 6A). It is unclear why SAP genomic DNA, with the same modifications as TB37, should be digested more weakly by His_6_-BrxU compared to the impact on TB37 genomic DNA (Fig. 6A and B). Unknown variables in digesting the full genomic DNAs (Fig. 6A and B) could include the impact of slight variation in genome size, differences in modification levels, and the different genome sequences and therefore context dependence of the modifications. Nevertheless, this experiment extends the substrate profile for BrxU further, to now include arabinosylated cytosines.

## DISCUSSION

In this study, we provide characterization of the second batch of Durham Collection phages, bringing the total to 24 coliphages: 12 with non-modified genomes (28) and a further 12 with modified genomes as presented herein. These phages were initially isolated from water sources in Durham, UK, by undergraduates as part of a final year workshop at Durham University. With this second batch, we provide fully annotated genomes, morphological descriptions via TEM, and detailed chemical analysis of the modifications present in their genomes. With this knowledge, we were able to demonstrate that BrxU, a GmrSD family Type IV restriction enzyme, has wider substrate specificity than previously reported.

Our aim has been to produce a curated set of coliphages to aid labs in performing standardized and comparable experiments. The 12 additions include 1 *Krischvirus*, 1 *Mosigvirus*, and 10 *Tequatrovirus* phages (Fig. 1 and Table 1). All have genomes between 160 and 170 kb, and all have myoviral morphology (Fig. 1). The *Krischvirus* SAP is related to RB49 (58); the *Mosigvirus* NP is related to RB69; and the *Tequatrovirus* phages are related to T-even phages (Fig. 2 and Fig. S1). As all these phages were sensitive to BrxU, we knew that they had modified bases in their DNA (14). The resulting chemical analysis identified diverse modifications (Fig. 3) that divided the phages into four groups (Fig. 4). Coupling the genome sequences to information from HPLC and MS analysis allowed us to identify likely pathways for generating the observed modifications (Fig. 5). The *Tequatrovirus* phages matched known routes that require generation of the 5-hmdCMP precursors prior to phosphorylation and replicative incorporation, then subsequent decoration by DNA glycosyltransferases. Curiously, despite the added bonus of having genomic data on TB37, a non-modified variant of SAP, it was not possible to identify likely modification pathways for *Krischvirus* phages, and this will be the topic of a separate study. Importantly, while completing this work, multiple studies examined the modification pathways for arabinosylation, such as those found in *Mosigvirus* phages RB69 and NP. RB69 was originally considered to have an arabinose modification linked through a hydroxymethyl group. New evidence indicates that the linkage for arabinosylation is through an ester generated using a 5′ hydroxy group (44, 45). Though we have been able to pinpoint likely biosynthetic genes in the *Mosigvirus* NP (Fig. 5), the full pathway has not yet been characterized, and it is unclear what the arabinose precursor might be. We detected the addition of one and two arabinose sugars to NP, and it is worth noting that other phages have been identified with up to three linked arabinose moieties (44, 45).

Many modifications have been identified in phage genomes (38, 39). No doubt, many biological roles will be discovered, but primary among them will be as a means to protect from the most widespread means of bacteriophage defense: restriction enzymes. Though Type I, II, and III restriction enzymes can be blocked by DNA modifications, Type IV or “modification-dependent” restriction enzymes specifically recognize and degrade DNA with modifications. One such family of Type IV enzymes is GmrSD, which includes BrxU. We previously demonstrated that BrxU could recognize and cleave multiple modifications, with a preference for 5-glc-mdC over 5-methyl-2′-deoxycytidine (5-mdC) over 5-hmdC (14, 46). Armed with knowledge of the wider range of substrates in the Durham Collection, we demonstrated that BrxU can also cleave 5-ara-dC and 5-ara-ara-dC modifications (Fig. 6). The first characterized bipartite GmrS-GmrD enzyme from *Escherichia coli* prophage CT596 acted on 5-glc-mdC but not 5-hmdC (47). Recently discovered TvgA-TvgB similarly cuts 5-glc-mdC but not 5-hmdC (59). Another single-chain GmrSD homolog, Eco94GmrSD, was shown to act on 5-glc-mdC and 5-hmdC but was not tested against 5-mdC (60). From the examined cases, it appears that fused single-chain GmrSD homologs might have a broader substrate range than the two gene homologs, but until a panel is assembled and tested against a standardized set of substrates (for instance, including those from the Durham Collection), this will remain unclear. Furthermore, as we do not yet have structural information on substrate recognition by BrxU, it is unclear for now why such a range of substrates can be accommodated.

The Durham Collection provides an additional set of characterized coliphages for research and discovery. These phages can be used to study phage-host interactions and will aid modeling and predictions of phage infections. In time, this collection may also contribute to clinical efforts towards promoting and effectively deploying phage therapy.

## MATERIALS AND METHODS

### Bacterial strains and culture conditions

*E. coli* strain DH5α was sourced from Invitrogen. *E. coli* ER2566 was sourced from New England Biolabs (61). All strains were grown at 37°C, either on agar plates or shaking at 150 rpm for liquid cultures. 2× YT was used as the standard growth media for liquid cultures, and Luria broth (LB) was supplemented with 0.35% wt/vol or 1.5% wt/vol agar for semisolid and solid agar plates, respectively. Growth was monitored using a spectrophotometer (WPA Biowave C08000) measuring optical density at 600 nm. When necessary, growth media were supplemented with ampicillin (100 µg/mL), D-glucose (0.2% [wt/vol]), or L-arabinose (0.1% [w/v]).

### Use of coliphages

The Durham Collection of phages was initially harvested and isolated by undergraduates as previously described (14). More details are available in Table 1 and Table S1. To make lysates, 10 μL of phage dilution was mixed with 200 μL of *E. coli* DH5α overnight culture and mixed with 4 mL of sterile semisolid “top” LB agar (0.35% agar) in a sterile plastic bijou. Samples were poured onto solid LB agar plates (1.5% agar) and incubated overnight at 37°C. Plates showing a confluent lawn of plaques were chosen for lysate preparations, and the semisolid agar layer was scraped off into 3 mL of phage buffer. Five hundred microliters of chloroform was added, and samples were vigorously vortexed and incubated for 30 min at 4°C. Samples were centrifuged at 4,000 × *g* for 20 min at 4°C, and the supernatant was carefully transferred to a sterile glass bijou. One hundred microliters of chloroform was added, and lysates were kept at 4°C for long-term storage.

### DNA isolation and manipulation

Phage genomic DNA was extracted using phenol-chloroform extraction methods. Briefly, 450 µL of phage lysate was incubated with 4.5 U DNase I (ThermoFisher Scientific) and 2 µL of 20 mg/mL RNase I (NEB) at 37°C for 30 min with shaking. Proteinase K (2.25 µL of 20 mg/mL stock [ThermoFisher Scientific]) and 10% SDS were added to a final concentration of 0.5% before incubation at 37°C for 30 min. The sample was then resuspended in 500 μL of 24:24:1 (vol/vol) phenol:chloroform:isoamyl alcohol (PCI) before centrifugation at 4°C for 5 min at 16,000 × *g*. The aqueous layer was transferred to a new tube; PCI treatment was repeated; and the subsequent aqueous layer was transferred to a new tube before treatment with 500 μL 24:1 (vol/vol) chloroform:isoamyl alcohol and centrifugation as above. The aqueous layer was transferred to a new tube and incubated with 45 µL 3 M sodium acetate pH 5.2 and 500 µL isopropanol for 15 min at room temperature. DNA was pelleted by centrifugation at 4°C for 20 min at 16,000 × *g*. The pellet was washed twice with 70% ethanol before being left to soak overnight at 4°C in EB (10 mM Tris-HCl 0.1 mM EDTA, pH 8.5), TE (10 mM Tris-base 1 mM EDTA, pH 8.0), or MilliQ dH_2_O. The integrity of gDNA was assessed via 0.75% agarose gels, and quantification was undertaken via NanoDrop One (ThermoFisher Scientific). In some cases, a Phage DNA Isolation kit (Norgen BioTek Corp) was used, as per the manufacturer’s instructions.

### Electron microscopy

High-titer phage lysates were applied to glow-discharged copper grids for 45 s before three successive washes in MilliQ, followed by staining in 2% phosphotungstic acid. Excess stain was removed by blotting before imaging via transmission electron microscopy, using a Hitachi H7600 operated at 100 kV accelerating voltage, equipped with an EMSIS Xarosa camera, and assessing images using Radius.

### Phage genome sequencing and annotation

Extracted phage gDNA was sequenced either via Illumina NovaSeq 6000 (phages BGP, EL, Geo, NP, NR1, SAP, and Solly) at MicrobesNG, or via Illumina MiSeq (phages CP, EH2, and TB37) or Illumina NextSeq (phages QOTSP and Some) at New England Biolabs. MicrobesNG genomic DNA libraries were prepared using the Nextera XT Library Prep Kit (Illumina, San Diego, USA) following the manufacturer’s protocol with the following modifications: input DNA was increased twofold, and PCR elongation time was increased to 45 s. DNA quantification and library preparation were carried out on a Hamilton Microlab STAR automated liquid handling system (Hamilton Bonaduz AG, Switzerland). Pooled libraries were quantified using the Kapa Biosystems Library Quantification Kit for Illumina. Libraries were sequenced using Illumina sequencers (HiSeq/NovaSeq) and a 250 bp paired-end protocol. Reads were adapter-trimmed using Trimmomatic 0.30 with a sliding window quality cutoff of Q15 (62). *De novo* assembly was performed on samples using SPAdes (v3.7) (63) with default settings, and contigs were annotated using Prokka 1.11 (64). For sequencing by Illumina MiSeq and Illumina NextSeq, the DNA was first sheared to 350 bp in TE buffer using a Covaris ME220 AFA ultrasonication system and microTUBE-130 AFA Fiber sample tubes. Library preparation was performed using the NEBNext Ultra II DNA Library Prep Kit for Illumina (NEB E7645) indexed with NEBNext Multiplex Oligos for Illumina (NEB E7335), amplified using NEBNext Ultra II Q5 Master Mix (four or five PCR cycles, NEB M0544S), and the library pool was sequenced on the Illumina MiSeq or on an Illumina NextSeq in paired-end reads of 150 or 76 bp. Sequencing reads were processed to trim off adaptors using Trim Galore (v0.6.4) (https://www.bioinformatics.babraham.ac.uk/projects/trim_galore/) using the paired option. Trimmed reads were used for performing the genome *de novo* assembly using MEGAHIT (v1.2.9) (65) with default settings.

Phage genome assemblies were queried against the core nucleotide database for highly similar sequences using BLAST-N (National Institute of Health). The highest-scoring alignments were analyzed by pairwise comparison using the Artemis Comparison Tool (66), and genomes were rearranged using SnapGene (v7.0.1). Rearranged genome assemblies were assessed using the BV-BRC PHANOTATE pipeline (67), available at https://github.com/deprekate/PHANOTATE, to identify open reading frames, tRNA genes, and terminators. Hypothetical protein identities were assigned according to CDS coverage and E-values from the workflow results. For predicted proteins where no significant similarity was found, InterPro (68) was used to try and assign a protein family identity. The sample was registered with the ENA, and the annotated genomes were validated and submitted using the Webin command line submission interface (v3.0.1–4.4.0). Accession numbers are available in Table 1 and Table S1.

### Phage taxonomy determination and variant calling

Sequences of the modified Durham phages were compared against a RefSeq database using mash dist (v2.3) (using the compressed sketch database at https://gembox.cbcb.umd.edu/mash/refseq.genomes.k21s1000.msh on 6 August 2023).

Complete genomes of phages belonging to the *Straboviridae* family were downloaded using the Entrez utilities (v16.2) (69) (*n* = 882). Genomes were compared and clustered using fastANI (v1.33) (70) and ANIClustermap (https://github.com/moshi4/ANIclustermap/blob/main/CITATION.cff) to eliminate redundancy and subset the data set to <200 genomes for input on VIPTree. One representative sequence from each cluster (Table S3) was chosen. The data set was curated to ensure that T-even phages T2, T4, and T6 and phages RB49 and RB69 were chosen as representatives of their respective sequence clusters.

The phylogenetic relationship between the curated *Straboviridae* data set and the sequence of the 12 modified Durham phages was determined using the VIPTree server (51), using the “only query” option. The taxonomic information for the curated *Straboviridae* data set was obtained with the use of Entrez utilities (v16.2. Trees were annotated using iTOL (71).

Variant calling was performed by using Snippy (v4.6.0) (https://github.com/tseemann/snippy). Comparisons were performed using complete genomes and the “--ctgs” option.

### Analysis of DNA modifications

Approximately 1 µg of phage DNA was treated with the Nucleoside Digestion Mix (NEB M0649) according to the manufacturer’s protocol at 37°C for >2 h. The resulting mixture of nucleosides was filtered through a hydrophilic PTFE 0.2 µm centrifugal filter, and the filtrate was subjected to reverse-phase HPLC-MS for nucleoside separation and detection. The HPLC-MS was performed on an Agilent 1290 Infinity II UHPLC-MS system equipped with a G7117 Diode Array Detector and an LC/MSD XT G6135 Single Quadrupole Mass Detector. A Waters XSelect HSS T3 C18 column (4.6 × 150 mm, 3 µm particle size) was used for the chromatography with the instrument, in which the methods were operated at a flow rate of 0.6 mL/min with a binary linear gradient mobile phase consisting of 10 mM ammonium acetate (pH 4.5) and methanol. The course of chromatography was monitored at 260 nm. Mass spectrometry was operated in both positive (+ESI) and negative (−ESI) electrospray ionization modes, with a capillary voltage of 2,500 V, a fragmentor voltage of 70 V, and a mass range of *m*/*z* from 100 to 1000 in both modes. Agilent ChemStation software was used for the primary LC-MS data processing. ChemStation-produced chromatograms were further annotated in Adobe Illustrator.

### Protein expression and purification

Production of His_6_-BrxU was performed as described using plasmid pTRB519 (14).

### BrxU assays

His_6_-BrxU titration assays were set up with 1 mM ATP, 1× BMG buffer (20 mM Tris-HCl, pH 7.5, 50 mM CH_3_COOK, and 10 mM MgSO_4_), and 100 ng of phage gDNA. Reactions were carried out at 37°C for 15 min. Timepoint assays were set up under the same conditions, but reactions were carried out for up to 30 min. Negative controls used ATPγS or nuclease-free water in place of ATP. All reactions were terminated by incubating at 75°C for 10 min.

Agarose gels comprised 0.8% agarose containing 0.004% (vol/vol) ethidium bromide and were resolved at 80 V for 45 min in 1× TAE buffer. All gels were run in triplicate and visualized with BioRad Image Lab software. The remaining substrate was quantified by comparison with the no ATP control using FIJI ImageJ.

## Data Availability

Annotated phage genomes are available via GenBank using the accession numbers listed in Table 1 and Table S1. All other data needed to evaluate the conclusions in the paper are present in the paper and/or supplemental material.

## SUPPLEMENTAL MATERIAL

Supplemental figures aem.00810-26-s0001.pdf Fig. S1 to S3.

Table S1 aem.00810-26-s0002.xlsx Full modified phage collection and submission details.

Table S2 aem.00810-26-s0003.xlsx CheckV analysis of phage genomes.

Table S3 aem.00810-26-s0004.xlsx Taxonomy of related phages.

Table S4 aem.00810-26-s0005.xlsx Straboviridae representatives plus custom additions.

Table S5 aem.00810-26-s0006.xlsx SNIPPY analysis of SAP vs TB34.

ASM does not own the copyrights to Supplemental Material that may be linked to, or accessed through, an article. The authors have granted ASM a non-exclusive, world-wide license to publish the Supplemental Material files. Please contact the corresponding author directly for reuse.
