# Supplementary material for: DNA modifications of Durham Collection phages and promiscuity of GmrSD-family Type IV restriction enzyme BrxU
Source: Appl Environ Microbiol. 2026 Jun 12;92(7):e00810-26. doi: 10.1128/aem.00810-26 (PMC13390438; doi:10.1128/aem.00810-26)
Supplement: Supplemental figures — Fig. S1 to S3. [file aem.00810-26-s0001.pdf]

Supplementary Materials

DNA-modifications of Durham Collection phages and promiscuity of GmrSD-family Type IV restriction enzyme BrxU

Jennifer J. Readshaw[a], Abigail Kelly[a], Yan-Jiun Lee[b], Giuseppina Mariano[c], Liam P. Shaw[a,d], Peter Weigele[b], Tim R. Blower[a,b,§]

[a]Department of Biosciences, Durham University, Stockton Road, Durham, DH1 3LE, UK.

[b]New England Biolabs, 240 County Road, Ipswich, MA 01938, USA.

[c]School of Infection and Immunity, 120 University Place, University of Glasgow, Glasgow G12 8TA, UK.

[d]Department of Biology, University of Oxford, 11a Mansfield Road, Oxford, OX1 3SZ, UK.

[§]To whom correspondence may be addressed. Email: tblower@neb.com, timothy.blower@durham.ac.uk tel: +19783806631.

Supplementary Figures

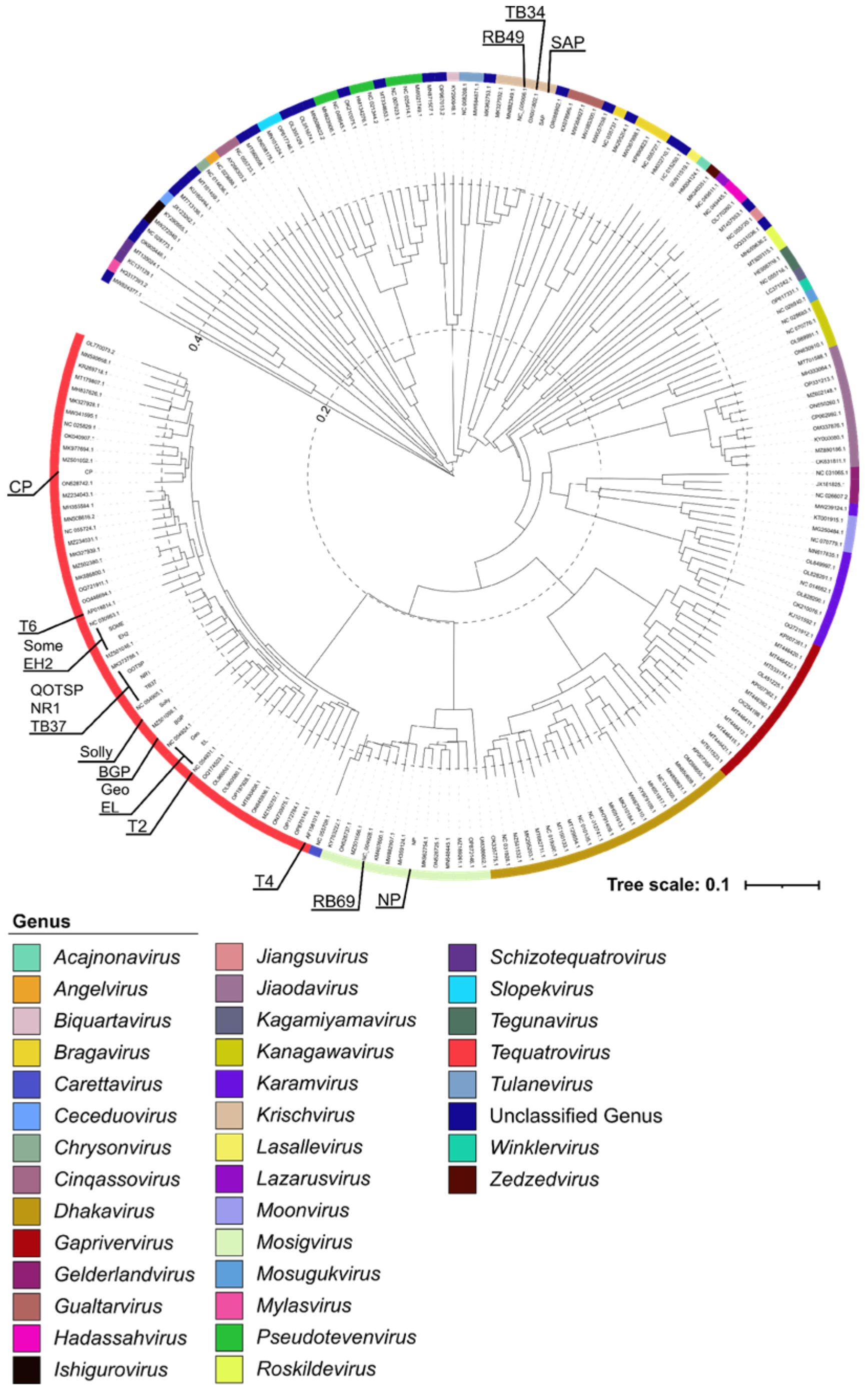

Fig. S1. Durham Collection phages with DNA modifications mostly cluster together. The 12 Durham phages were compared to representative *Caudoviricetes* phages T2, T4, T6, TB36, RB49 and RB69. The tree scale reported refers to the branch length metadata of the tree. Similarly, an internal scale of the tree, based on branch migration, is also shown. Coloured strips indicate the taxonomy of each branch at the genus level.

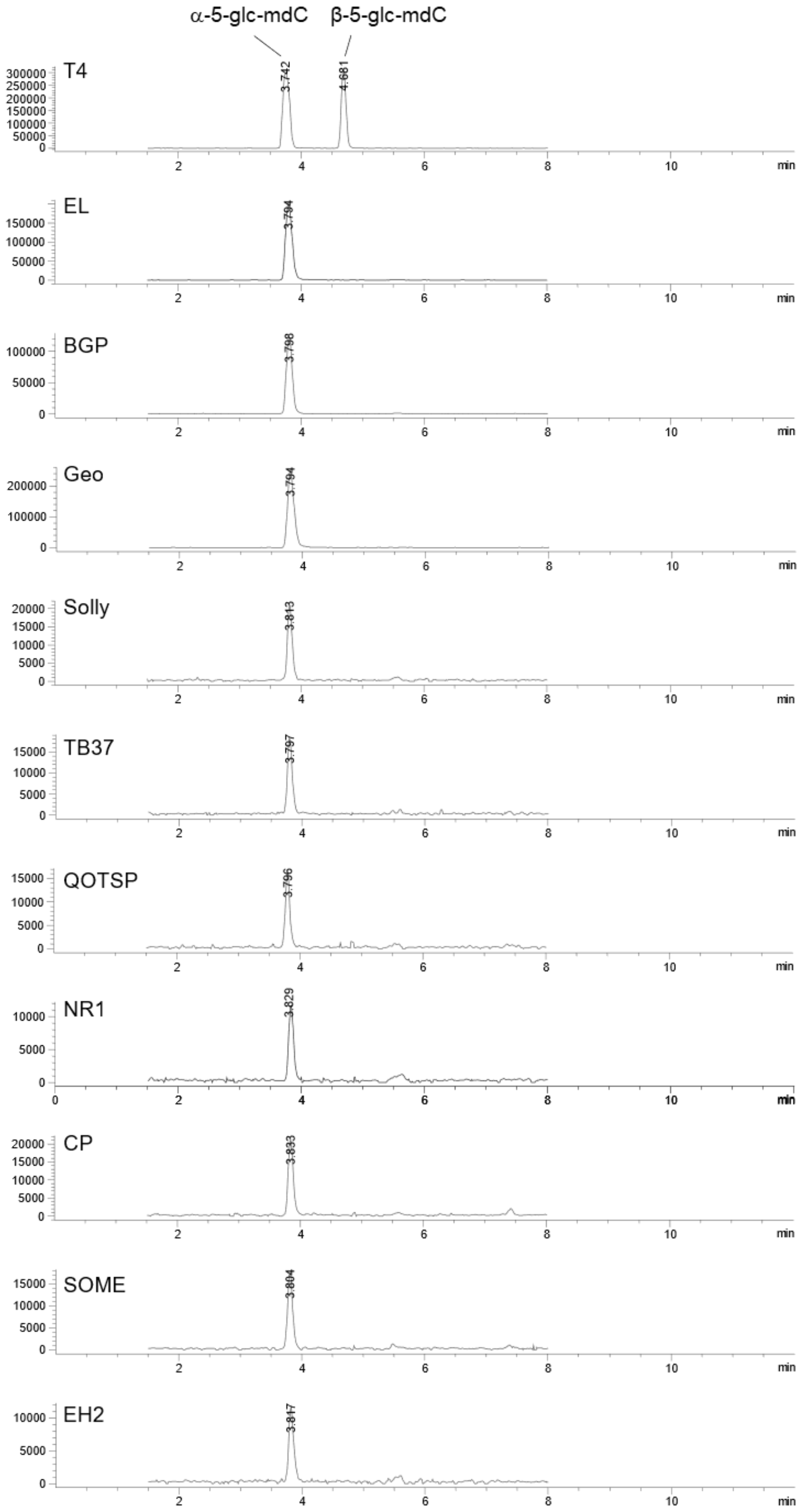

Fig. S2. LC-MS extracted-ion chromatography analysis of α-5-glucosylmethyl-2'-deoxycytidine (m/z 419) in the Durham Collection phages DNA. Hydrolysed genomic DNA from T4, which has both α- and β-5-glucosylmethyl-2'-deoxycytidine, is provided as the modification reference. Durham Collection phages contain α-5-glucosylmethyl-2'-deoxycytidine but not β-form.

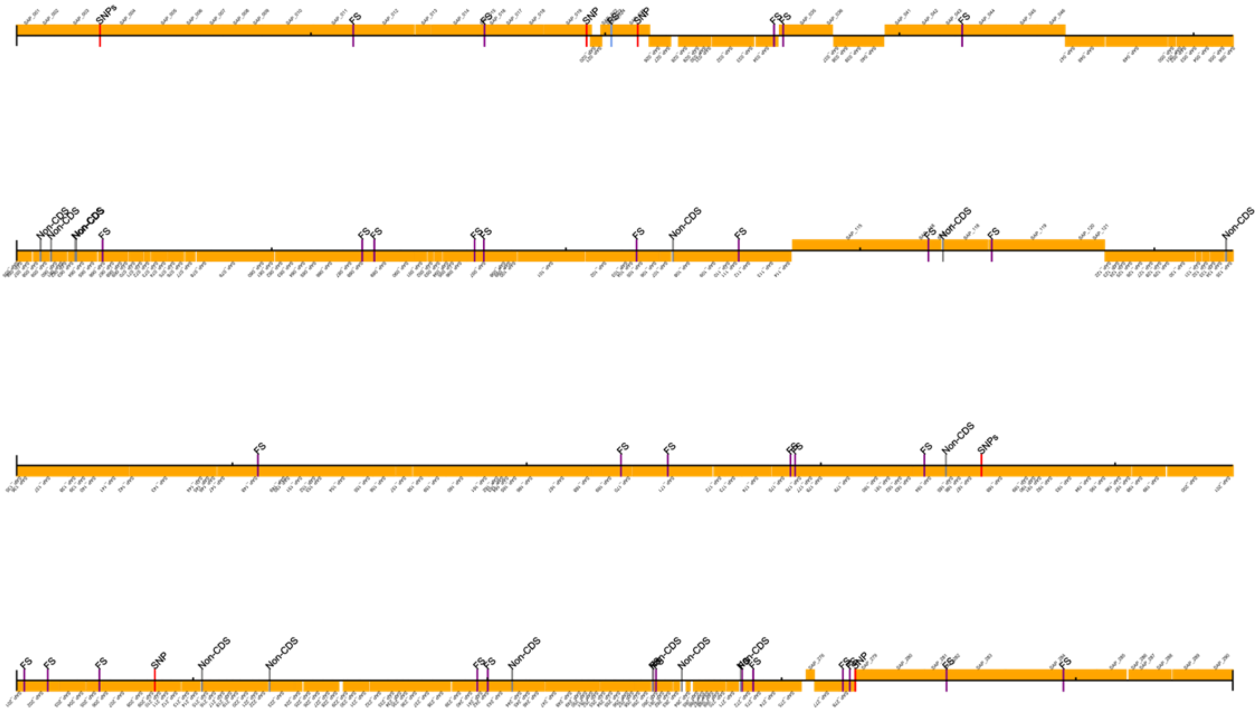

Fig. S3. SNIPPY analysis of SAP vs TB34. Positions of SNPs between TB34 and SAP are patterned on the SAP genome. For full details of SNP location and likely target gene, see **Table S4**.

Supplementary Tables

Provided as .xlsx files

Table S1. Full modified phage collection and submission details

Table S2. CheckV analysis of phage genomes

Table S3. Taxonomy of related phages

Table S4. Straboviridae representatives plus custom additions

Table S5. SNIPPY analysis of SAP vs TB34
